# Prevalence, molecular characterization, and prognosis of c-Met protein overexpression in a real-world cohort of patients with non-squamous non-small cell lung cancer

**DOI:** 10.2340/1651-226X.2025.44344

**Published:** 2025-11-18

**Authors:** Jair Bar, Mona H. Cai, Yookyung Christy Choi, Shobhit Baijal, Weilong Zhao, Alexander Liede, Athan Vasilopoulos, Lisa Roberts-Rapp, Fang Jiang, Archana Simmons, Christine Ratajczak, Shun Lu, Peter J. Ansell, D. Ross Camidge

**Affiliations:** a Jusidman Cancer Center, Sheba Medical Center, Ramat Gan, Israel; b The Gray Faculty of Medical and Health Sciences, Tel Aviv University, Tel Aviv, Israel; c AbbVie, Inc., North Chicago, IL, USA; d University Hospitals Birmingham NHS Foundation Trust, Birmingham, UK; e Shanghai Chest Hospital, Shanghai Jiao Tong University, Shanghai, China; f University of Colorado Cancer Center, Aurora, CO, USA

**Keywords:** NSCLC, real-world data, c-Met protein overexpression, *MET* amplification, Teliso-V

## Abstract

**Background and purpose:**

c-Met (also known as MET) protein is encoded by the *MET* proto-oncogene. In non-small cell lung cancer (NSCLC), c-Met protein overexpression (OE) drives tumorigenesis and is a therapeutic target, given recent US Food and Drug Administration approval of telisotuzumab vedotin-tllv. This retrospective analysis of tumor samples and clinical data from real-world patients with non-squamous NSCLC characterized the prevalence of c-Met protein OE, its association with messenger ribonucleic acid (mRNA) expression, *MET* gene amplification, programmed-death ligand 1 (PD-L1) expression, and its impact on prognosis.

**Patients and methods:**

A patient cohort was selected for manual abstraction of clinical data from electronic health records. Patients were selected based on the availability of sufficient remnant tissue for biomarker analyses, including c-Met immunohistochemistry (IHC). Comparative assessments were conducted for c-Met protein expression by IHC, *MET* gene amplification, mRNA expression, and PD-L1 expression levels by IHC.

**Results:**

In total, 305 and 84 patients were included in the biomarker prevalence and outcome analyses, respectively. Overall, c-Met protein OE was detected in 25% of tissue samples. Of the 212 samples with fluorescence in situ hybridization data, *MET* amplification was seen in 9%. Concordance of c-Met protein OE with *MET* mRNA levels was observed with area under the concentration-time curve values of 0.738 and 0.736 in MET OE or MET high OE, respectively, using Receiver Operating Characteristic analysis. c-Met protein OE was associated with poor prognosis (unadjusted hazard ratio for death of 2.04).

**Interpretation:**

These data suggest that c-Met protein OE is associated with *MET* mRNA expression, shows limited overlap with other *MET* aberrations, and may be linked to poor prognosis in NSCLC.

## Introduction

c-Met (also known as MET) protein is a receptor tyrosine kinase encoded by the *MET* proto-oncogene. Under normal physiological conditions, hepatocyte growth factor (HGF) binds to its receptor, c-Met, resulting in the activation of multiple intracellular pathways that regulate a variety of cellular functions including cell proliferation, scattering and motility, morphogenesis, and protection from apoptosis [1–3]. The c-Met/HGF signaling pathway is dysregulated in cancer and correlates with abnormal tumor proliferation and survival, tumor invasion, progression, and metastasis [4–6]. In non-small cell lung cancer (NSCLC), the *MET-*associated molecular mechanisms that drive tumorigenesis are c-Met protein overexpression (OE), *MET* amplification, and splice-site mutations leading to *MET* exon 14 skipping mutations. c-Met protein OE is the most common of these aberrations and can occur independently of *MET* amplification and *MET* exon 14 skipping mutations [7–9]. As such, c-Met protein is a potential therapeutic target for antibody-drug conjugate (ADC) therapies in c-Met protein–overexpressing NSCLC [10–12].

ADCs allow for precise targeting of tumor surface antigens to deliver a toxic payload directly to cancer cells. One such agent is telisotuzumab vedotin-tllv (Teliso-V; ABBV-399), composed of the c-Met protein–directed monoclonal antibody telisotuzumab conjugated to a potent monomethyl auristatin E cytotoxin. On May 14, 2025, Teliso-V was granted accelerated approval by the US Food and Drug Administration (FDA) for the treatment of adult patients with locally advanced or metastatic, non-squamous NSCLC with high c-Met protein OE who have received a prior systemic therapy. High c-Met protein OE is defined as ≥ 50% of tumor cells with strong (3+) staining as determined by a simultaneously FDA-approved companion diagnostic test, the anti-MET antibody clone SP44 assay (Roche Diagnostics) [13]. The approval was based on positive results from the single-arm phase 2 LUMINOSITY trial (NCT03539536) of Teliso-V monotherapy in patients with previously treated, c-Met protein–overexpressing, epidermal growth factor receptor (*EGFR*) wild-type (WT) advanced/metastatic non-squamous NSCLC [14]. The trial demonstrated a tolerable safety profile and durable responses, with an objective response rate of 28.6% (34.6% in patients with high c-Met protein OE [i.e. c-Met high]), median duration of response of 8.3 months (9.0 months in c-Met high), and median overall survival of 14.5 months (14.6 months in c-Met high) [14].

With the recent approval of Teliso-V, and growing evidence that c-Met protein OE is a biomarker with clinical significance, further characterization of c-Met protein–overexpressing NSCLC is warranted. Establishing the prevalence of c-Met protein OE, its overlap with other known NSCLC biomarkers and *MET* genomic alterations (e.g. *MET* amplification), and its relevance to prognosis are crucial to fully understanding the potential benefits of c-Met–targeting ADCs in advanced NSCLC.

Herein, we present results of a retrospective analysis of tumor samples and clinical data from a real-world cohort of patients with NSCLC. The selection criteria were designed to reflect, as closely as possible, the patient population enrolled in the LUMINOSITY study. The objective was to characterize the prevalence of c-Met protein OE, its association with messenger ribonucleic acid (mRNA) expression, *MET* gene amplification, and programmed-death ligand 1 (PD-L1) expression, and its impact on real-world prognosis.

## Methods

### Ethics

This retrospective analysis of patient tumor specimens and clinical data was reviewed and approved by the institutional review board at the City of Hope National Medical Center.

### Patients

Patients with non-squamous NSCLC seeking care at the City of Hope National Medical Center and with available tumor biopsy samples were selected for manual abstraction of clinical data from electronic health records. Tumor samples were collected in or after 2010 and assessed for adequate remaining tissue to conduct biomarker analysis. This included the ability to generate 10 tissue slides at 4-µM thickness, with a minimum of 20% tumor content.

### Biomarker analysis

#### c-Met protein expression

Prior to testing, samples underwent a pathology review to ensure at least 100 viable tumor cells were present. c-Met protein expression levels were determined using an investigational-use-only immunohistochemistry (IHC) assay utilizing anti-MET antibody clone SP44 (Roche Diagnostics) on a BenchMark ULTRA instrument. Thresholds chosen to define c-Met protein OE were those used in the LUMINOSITY study [14]. c-Met positivity was defined as ≥ 25% tumor cells with 3+ staining intensity. Intermediate and high c-Met protein OE by IHC were defined as ≥ 25 to < 50%, and ≥ 50% tumor cells with 3+ staining intensity, respectively.

#### *MET* gene amplification

Fluorescence in situ hybridization (FISH) signal counts were determined in the targeted tumor areas using two distinct DNA probes – a chromosome enumeration probe (CEP) that hybridizes to a centromere of chromosome 7, and a second probe that hybridizes to the *MET* gene, manufactured by Abbott Molecular. The threshold for gene amplification was defined as a *MET*/CEP7 FISH signal count ratio ≥ 1.8. Chi-squared analysis was performed to determine association with c-Met protein OE.

#### mRNA sequencing

mRNA sequencing was performed using Personalis ImmunoID NeXT^TM^ ribonucleic acid (RNA) sequencing (RNA-Seq) (Personalis NeXT^TM^ Transcriptome^TM^) following the manufacturers’ recommendations.

#### PD-L1 expression

PD-L1 expression was assessed using the PD-L1 IHC 22C3 pharmDx assay. Tumor Proportion Score (TPS) was used to categorize PD-L1 levels as high (≥ 50% of tumor cells positive), low (1–49%), or negative (< 1%). Chi-squared analysis was performed to determine association with c-Met protein OE.

#### Prognostic value

To evaluate the prognostic relevance of c-Met protein expression, a separate analysis was conducted in a subset of patients representative of those enrolled in the LUMINOSITY clinical study [14], which included patients with *EGFR* WT NSCLC and c-Met protein OE (≥ 25% tumor cells exhibiting strong 3+ staining intensity). To accomplish this, the analysis was limited to patients with stage IV non-squamous, metastatic NSCLC seeking care at City of Hope National Medical Center with adequate tissue for c-Met protein IHC. Furthermore, given immune checkpoint inhibitor (ICI) therapies were first approved in the metastatic setting in 2015, this analysis was restricted to 2016 or later to capture patients who could have been treated with ICI-based therapies. Patients receiving first-line targeted therapy including tyrosine kinase inhibitors were excluded from the analysis. Clinical data were extracted from electronic health records, and unadjusted hazard ratios from first-line treatment initiation until death or censoring were used to determine outcomes; patients with c-Met protein OE were compared with patients without c-Met protein OE. Kaplan-Meier curves and unadjusted hazard ratios from first-line treatment initiation until death or censoring event were used to determine outcomes. Patients were censored at the clinical trial enrollment date, at another primary NSCLC diagnosis, or at the last follow-up (last contact date + 30 days). Hazard ratios and 95% confidence intervals were calculated. The latest follow-up for this cohort was in Quarter 1, 2023.

## Results

### Patients

**Figure 1:**
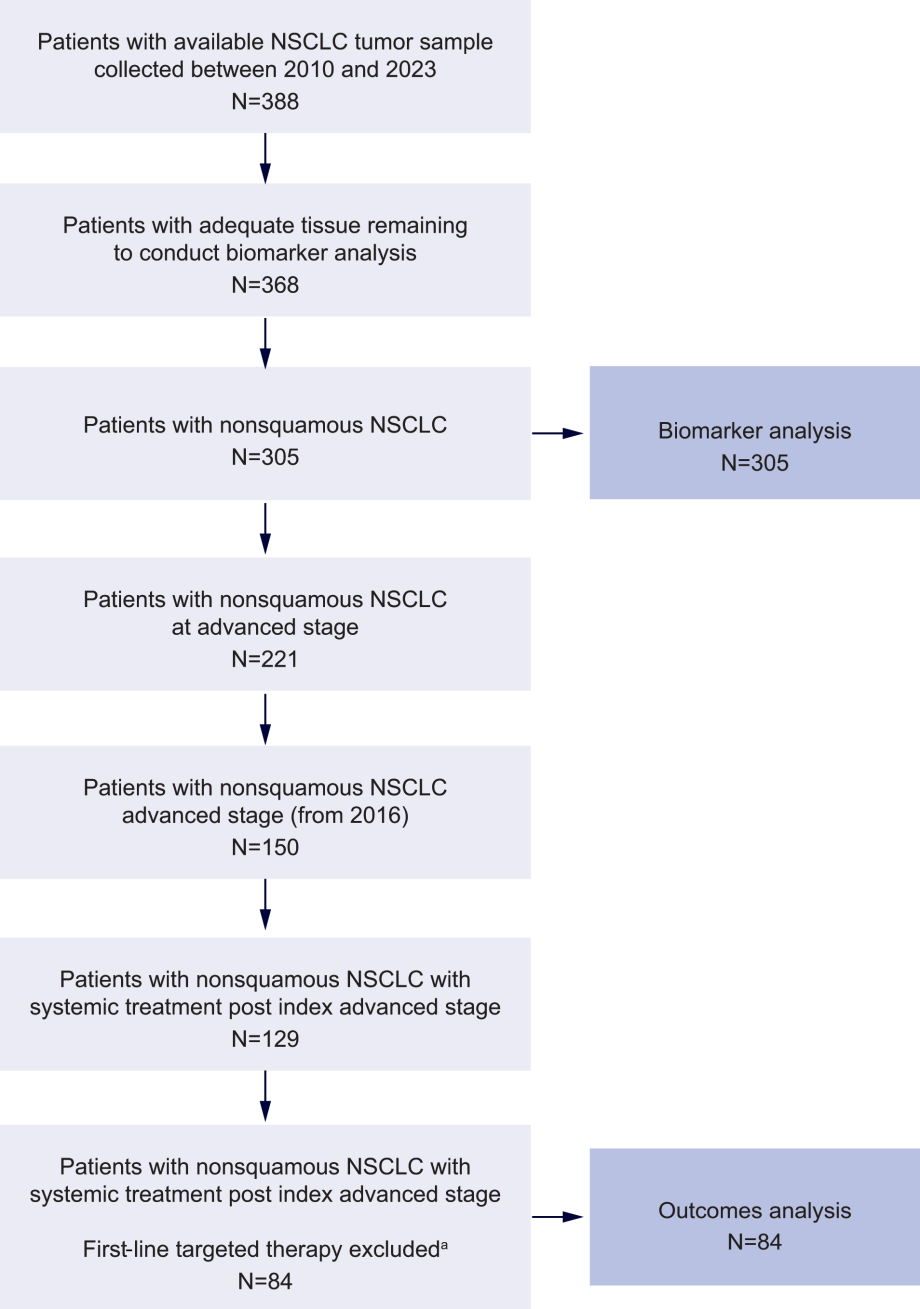
Summary of patient attrition. The index data is the earliest date of metastasis (or diagnosis date for patients diagnosed at stage 4). ^a^Excluded first-line targeted therapy class include EGFR inhibitor (*n* = 34), ALK inhibitor (*n* = 9), KRAS inhibitor (*n* = 1), BRAF inhibitor (*n* = 1). ALK: anaplastic lymphoma kinase; BRAF: B-Raf proto-oncogene, serine/threonine kinase; *EGFR*: epidermal growth factor receptor; KRAS: Kirsten rat sarcoma viral oncogene homolog; NSCLC: non-small cell lung cancer.

In total, 305 patients with non-squamous, metastatic NSCLC were included in the analysis of biomarker prevalence (Figure 1). The median age was 66 (range: 28–92) years, and 172 (56%) patients were female. Detailed demographics and baseline characteristics of the overall population are shown in Table 1. A subgroup of 84 patients diagnosed with stage IV disease in 2016 or later were included in the outcome analysis evaluating the impact of c-Met protein OE on prognosis.

**Table 1:** Demographics and baseline characteristics of the overall population of patients diagnosed with non-squamous NSCLC.

Characteristic	Overall (*N* = 305)	c-Met protein OE positive (*n* = 77)	c-Met protein OE negative (*n* = 228)
**c-Met protein status, *n* (%)**			
High	42 (14)	42 (55)	-
Intermediate	35 (11)	35 (45)	-
Negative	228 (75)	-	228 (100)
**Median age, years (range)**	66 (28–92)	65.7 (33–85)	66.1 (28–92)
**Female, *n* (%)**	172 (56)	43 (56)	129 (57)
**Race, *n* (%)**			
Asian	61 (20)	18 (23)	43 (19)
Black	23 (8)	8 (10)	15 (7)
White	212 (79)	51 (66)	161 (71)
Other/unknown	9 (3)	0	9 (4)
**Ethnicity, *n* (%)**			
Hispanic/Latino	27 (9)	7 (9)	20 (9)
Not Hispanic/Latino	272 (89)	70 (91)	202 (89)
Unknown	6 (2)	0	6 (3)
**Stage at diagnosis, *n* (%)**			
I–III	160 (52)	35 (45)	125 (55)
IV	111 (36)	34 (44)	77 (34)
Unknown	34 (11)	8 (10)	26 (11)
**Smoking status, *n* (%)**			
Ever smoker	207 (68)	44 (57)	163 (71)
Never smoker	98 (32)	33 (43)	65 (29)
***EGFR* mutation, *n* (%)**			
Yes	21 (7)	10 (13)	11 (5)
No/Unknown	284 (93)	67 (87)	217 (95)
***KRAS* mutation, *n* (%)**			
Yes	50 (16)	7 (9)	43 (19)
*KRAS* pG12C	19 (6)	3 (4)	16 (7)
No	255 (84)	70 (91)	185 (81)

c-Met protein OE positive is defined as ≥ 25% tumor cells with 3+ intensity; high OE as ≥ 50% tumor cells with 3+ staining intensity; intermediate OE as ≥ 25 to < 50% tumor cells with 3+ staining intensity. *EGFR*: epidermal growth factor receptor; KRAS: Kirsten rat sarcoma viral oncogene homolog; NSCLC: non-small cell lung cancer; OE: overexpression.

### c-Met protein OE

c-Met protein OE was detected in 25% of patient tumor specimens (77/305), with 42 (14%) and 35 (11%) patients showing high and intermediate c-Met protein OE, respectively. Among patients with c-Met protein positivity per IHC, 55% had high OE. There was no association between a history of smoking and c-Met protein OE – 21% (44/207) of ever smokers and 34% (33/98) of never smokers were positive for c-Met protein OE. Of all tumor samples, 61 were biopsies and 244 were surgical specimens. The prevalence of c-Met protein OE was 33% in biopsy samples and 23% in resection specimens. Samples were taken from diverse anatomic locations. When grouping by lung vs non-lung, the c-Met protein OE prevalence was 24% (*n* = 204) in samples taken from the lung and 28% (*n* = 101) in samples taken from non-lung sites.

### Association with MET gene amplification

FISH results for *MET* gene amplification were available for 212 tissue samples. Of these, 19/212 (9%) were *MET* amplified. In patients with c-Met protein–overexpressing non-squamous NSCLC, *MET* was amplified in 18% of samples tested (*n* = 9/49; c-Met high, 21% [6/28]; c-Met intermediate, 14% [3/21]). Among patients negative for c-Met OE, the prevalence of *MET* amplification was 6% (*n* = 10/163). A chi-square test yielded a P value of 0.02, indicating a statistically significant association between c-Met protein OE and *MET* gene amplification. Despite the association, these results indicate that c-Met protein OE frequently occurs in the absence of *MET* gene amplification.

Among samples tested for *MET* gene amplification, 47 were biopsies and 165 were surgical specimens. The *MET* amplification rate was 6 and 10% in biopsy and resection specimens, respectively. However, caution should be taken in the interpretation of these results, given the low number of samples with *MET* amplification. When grouping by anatomic site, the *MET* amplification rate was 6% in samples taken from the lung (*n* = 141) and 14% in samples taken from non-lung sites (*n* = 71).

#### Concordance of c-Met protein OE by IHC and *MET* mRNA

**Figure 2:**
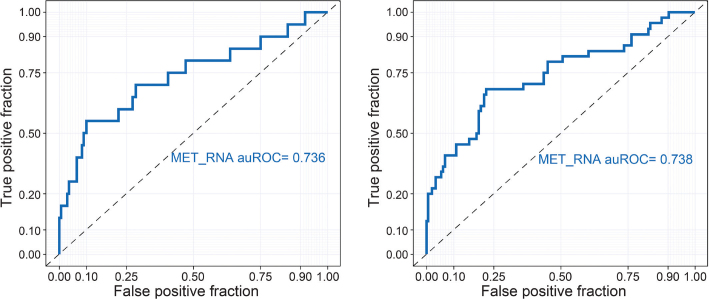
*MET* mRNA levels in c-Met protein overexpression-positive and -negative tumor specimens among patients with non-squamous NSCLC. ROC analysis was used to associate *MET* mRNA levels with c-Met protein OE. Left: ROC analysis evaluating MET mRNA expression vs c-Met protein overexpression by IHC in which c-Met protein OE positive is defined as ≥ 25% tumor cells with 3+ intensity. Right: ROC analysis evaluating MET mRNA expression vs high c-Met protein overexpression by IHC in which c-Met protein OE positive is defined as ≥ 50% tumor cells with 3+ intensity. ROC: Receiver Operating Characteristic; auROC: area under the ROC curve; NSCLC: non-small cell lung cancer; OE: overexpression; IHC: immunohistochemistry; mRNA: messenger ribonucleic acid..

A total of 188 patient samples were analyzed for concordance of c-Met protein OE with *MET* mRNA levels using RNA-Seq. Concordance of c-Met protein OE with *MET* mRNA levels was observed, as demonstrated by area under the concentration-time curve values of 0.738 and 0.736 in samples with c-Met protein OE or c-Met protein high OE, respectively, using Receiver Operating Characteristic analysis (Figure 2).

#### PD-L1 levels

**Figure 3:**
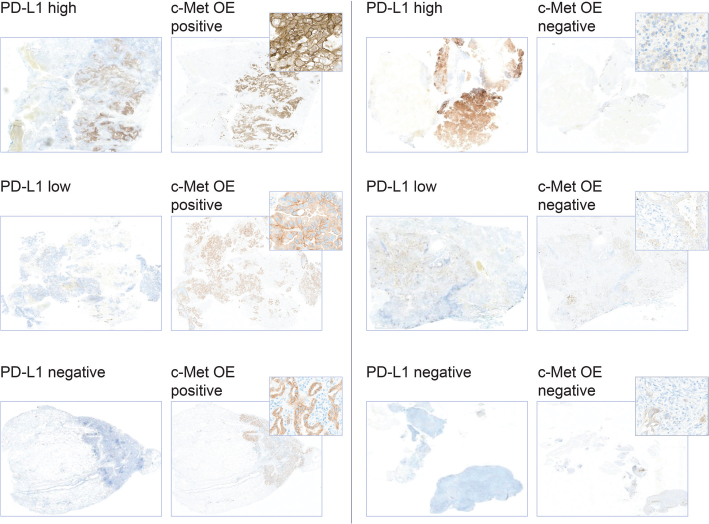
Examples of c-Met protein overexpression and PD-L1 staining categories for tumor specimens among patients with non-squamous NSCLC. PD-L1 high: ≥ 50% cells expressing PD-L1; PD-L1 low: 1–49% cells expressing PD-L1; PD-L1 negative: ≤ 1% cells expressing PD-L1. C-Met protein OE positive is defined as ≥ 25% tumor cells with 3+ intensity. NSCLC: non-small cell lung cancer; OE: overexpression; PD-L1: programmed-death ligand 1.

**Figure 4:**
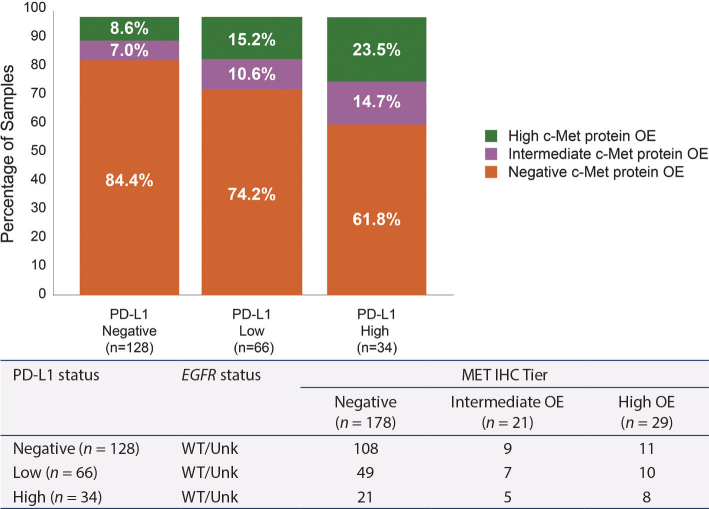
c-Met overexpression and PD-L1 levels among patients with non-squamous NSCLC^a^. ^a^Patients receiving targeted therapy as first-line treatment including tyrosine kinase inhibitors were excluded. Overall, *EGFR* status was unknown for 65 patients. PD-L1 high: ≥ 50% cells expressing PD-L1, PD-L1 low: 1–49% cells expressing PD-L1, PD-L1 negative: ≤ 1% cells expressing PD-L1. C-Met protein OE positive is defined as ≥ 25% tumor cells with 3+ intensity; high OE as ≥ 50% tumor cells with 3+ staining intensity, intermediate OE as ≥ 25 to < 50% tumor cells with 3+ staining intensity. *EGFR*: epidermal growth factor receptor; IHC: immunohistochemistry; NSCLC: non-small cell lung cancer; OE: overexpression; PD-L1: programmed-death ligand 1; Unk: unknown; WT: wild-type.

For the IHC analysis of PD-L1, results from 228 samples were available (21 patient samples were excluded due to confirmed *EGFR* mutation and 56 due to failed PD-L1 analysis). In this cohort, 56% of samples were PD-L1 negative, 29% were PD-L1 low, and 15% were PD-L1 high. c-Met protein IHC OE -positive and -negative cells were present across PD-L1 staining categories (Figure 3). Additionally, there was a trend of correlation between c-Met protein OE and PD-L1 IHC levels (chi-squared *P* value .06) (Figure 4). c-Met protein OE was observed in 16, 26, and 38% of PD-L1–negative, –low, and –high tumors, respectively.

#### *TROP2* mRNA levels

A total of 163 *EGFR* WT/unknown (Unk) non-squamous patient samples underwent all three assays, including c-Met protein IHC, PD-L1 IHC, and RNA-Seq. In these samples, the overlap of trophoblast cell-surface antigen 2 (*TROP2*) and *MET* mRNA expression (based on the RNA-Seq data) was analyzed to further characterize the anti–c-Met ADC target patient population and potential combination strategies with PD-1/PD-L1 inhibition and/or TROP2-targeted ADCs. The relative expression of *TROP2* and *MET* mRNA differed among PD-L1 expression categories. In patients with high PD-L1 expression, the median *MET* mRNA level was two-fold higher than the median level in PD-L1–negative samples. Median expression of *TROP2* mRNA was relatively consistent across PD-L1 expression categories, unlike *MET* mRNA, which tended to increase with higher PD-L1 IHC categories. In all settings, median *MET* mRNA level was higher than the median *TROP2* mRNA level (Supplemental Data Figure 1).

#### Impact of c-Met protein OE on prognosis

A subgroup of patients with *EGFR* WT, metastatic non-squamous NSCLC (*n* = 84) was evaluated to understand the influence of c-Met protein OE on prognosis (Figure 1). The baseline characteristics and demographics of these patients diagnosed with stage IV disease in 2016 or later are shown in Table 2. Approximately half the patients (52%) had received chemotherapy as first-line therapy; 26% had received chemotherapy plus ICI, and 21% had received ICI only as first-line therapy.

**Table 2:** Baseline characteristics of patients with metastatic non-squamous NSCLC included in the analysis of c-Met protein overexpression impact on prognosis (*N* = 84).

Characteristic	Overall (*N* = 84)	c-Met protein OE positive (*n* = 21)	c-Met protein OE negative (*n* = 63)
**Median age, years (IQR)**	67.5 (61–76)	66 (60–75)	65.5 (58–74.5)
**Female, *n* (%)**	46 (55)	11 (52)	53 (56)
**Stage at diagnosis, *n* (%)**			
I–III	39 (54)	12 (60)	27 (52)
IV	33 (46)	8 (40)	25 (48)
**Metastasis year, *n* (%)**			
2016–2017	28 (33)	2 (10)	26 (41)
2018–2019	28 (33)	6 (29)	22 (35)
2020–2022	17 (20)	8 (38)	9 (14)
2022–2023	11 (13)	5 (24)	6 (10)
**First line of therapy classification, *n* (%)**			
Chemotherapy	44 (52)	13 (62)	31 (49)
ICI	18 (21)	5 (24)	13 (21)
ICI plus chemotherapy	22 (26)	3 (14)	19 (30)
***KRAS* mutation, *n* (%)**			
Yes	22 (26)	3 (14)	19 (30)
*KRAS* pG12C	13 (15)	2 (9)	11 (17)
No	62 (74)	18 (86)	44 (70)

c-Met protein OE positive is defined as ≥ 25% tumor cells with 3+ intensity. ICI: immune checkpoint inhibitor; IQR: interquartile range; *KRAS*: Kirsten rat sarcoma viral oncogene homolog; NSCLC: non-small cell lung cancer; OE: overexpression.

**Figure 5:**
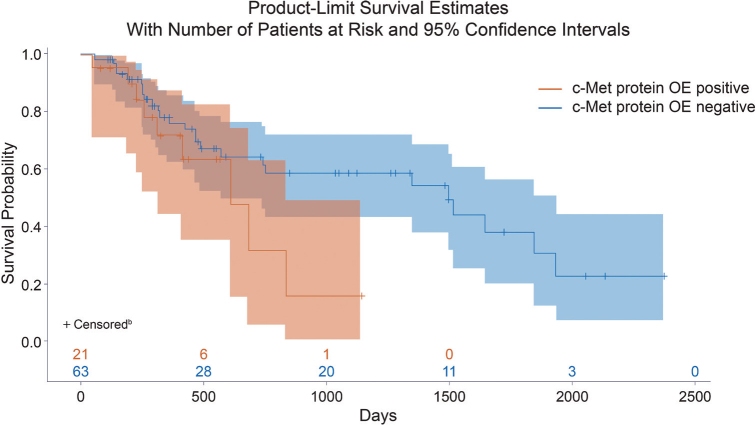
c-Met protein overexpression and survival probability in real-world patients. ^a^Patients receiving targeted therapy as first-line treatment including tyrosine kinase inhibitors were excluded. ^b^Patients were censored at clinical trial enrollment, last follow-up, or development of a new primary lung cancer, whichever occurred first. c-Met protein OE positive is defined as ≥ 25% tumor cells with 3+ intensity. OE: overexpression.

Overall, 21 (25% of this subgroup) patients had c-Met protein–overexpressing NSCLC. c-Met protein OE was associated with poor prognosis in these real-world patients; for patients with c-Met protein–overexpressing NSCLC versus no OE, the unadjusted hazard ratio for death was 2.04 (95% CI: 1.02, 4.10) (Figure 5).

## Discussion

Following the recent approval of Teliso-V, the first ADC approved for treating adults with locally advanced or metastatic non-squamous NSCLC exhibiting high c-Met protein OE, this study provides valuable insights into the prevalence of c-Met protein OE in a real-world patient cohort, and its impact on prognosis. Of note, this retrospective analysis of c-Met protein is the first to use the FDA-approved anti-MET antibody clone SP44 assay (Roche Diagnostics) for measuring c-Met protein levels.

The analysis indicated that 25% of patients had c-Met protein–overexpressing NSCLC, as defined by the cutoff used in the LUMINOSITY study [14]. This result is consistent with archival tissue observations in patients with non-squamous, *EGFR* WT NSCLC in the phase 2 LUMINOSITY trial, where the prevalence of c-Met protein OE was 22.1% [14]. Our study suggests that c-Met protein OE is independent of *MET* gene amplification, but is associated with *MET* RNA expression as evaluated by RNA-Seq. These data highlight the complexity of the relationship between c-Met protein OE, *MET* gene amplification, and *MET* RNA levels [11, 15–17]. In some cases, *MET* amplification may lead to increased *MET* RNA levels, resulting in higher c-Met protein expression; however, other regulatory mechanisms may also influence c-Met protein levels. High *MET* RNA levels without *MET* amplification occur if there is increased transcription of the *MET* gene, and conversely, *MET* amplification may not always result in elevated *MET* RNA levels if there are additional regulatory mechanisms that control mRNA stability or translation.

Focusing on the association between PD-L1 levels and c-Met protein OE, our analysis showed that OE was enriched in tumor samples with high PD-L1 levels. Furthermore, we observed a positive trend in c-Met protein OE positivity from low to high PD-L1, supporting the rationale of investigating c-Met protein–targeting ADCs in combination with PD-1/PD-L1 inhibitors for these tumors. Notably, when comparing *TROP2* mRNA expression to *MET* mRNA, *MET* mRNA levels were higher than *TROP2* mRNA in samples regardless of PD-L1 expression levels.

While c-Met protein OE has been associated with poorer outcomes in patients with surgically resected NSCLC [18, 19], the impact on prognosis in patients with advanced/metastatic NSCLC has been unclear. Our analysis indicates that c-Met protein OE is also associated with poorer survival outcomes in patients with advanced/metastatic NSCLC treated with standard-of-care treatments, including ICI.

Treatment options are limited for advanced/metastatic NSCLC. Recent advances have led to the development of targeted therapies for specific genomic alterations, transforming the treatment landscape for patients with targetable mutations. However, many patients with advanced disease have no detectable mutations, and there remains a high unmet need for effective treatments. ADCs represent a promising therapeutic approach, allowing the targeting of tumor cells expressing specific antigens, independently of oncogene driver mutations, and Teliso-V is the first ADC to be approved in this setting. Our results show that around 25% of patients with *EGFR* WT or unknown (Unk) non-squamous NSCLC overexpress c-Met protein, vs 3–5% of patients who harbor *MET* exon 14 skipping mutations or have *MET* gene amplification, strongly indicating that the development of treatments for c-Met protein–overexpressing NSCLC may improve outcomes for these patients. *EGFR* status was specifically evaluated, as it was a key exclusion criterion for Part 2 of the LUMINOSITY trial [14]. Understanding the interplay of other prominent NSCLC drivers, including Kirsten rat sarcoma viral oncogene homolog *(KRAS),* will be important. Data from the LUMINOSITY trial demonstrated that all 5 patients with *KRAS* G12C mutations demonstrated an objective response to treatment with Teliso-V [20]. A larger cohort will be required to better elucidate c-Met protein OE in the context of the broader NSCLC biomarker landscape. Furthermore, advancing our understanding of PD-L1 expression patterns and the molecular targets of emerging ADCs is critical to developing optimized diagnostic and therapeutic strategies for novel, next-generation ADCs.

This study has limitations associated with its retrospective design and reliance on real-world patient tumor specimens and clinical data. The overall patient cohort was relatively small, and data may have been incomplete or inconsistent, potentially affecting the accuracy of the analysis. Controlling for confounding variables is particularly challenging, especially when evaluating the prognostic significance of increased c-Met protein OE. Additionally, the prevalence of oncogenic drivers in NSCLC, including mutations in *EGFR, KRAS (*e.g. G12C*),* and B-Raf proto-oncogene, serine/threonine kinase (*BRAF*) V600E, as well as gene rearrangements involving anaplastic lymphoma kinase (*ALK*)*,* ROS proto-oncogene 1, receptor tyrosine kinase (*ROS1*), neurotrophic tyrosine receptor kinase (*NTRK*)*,* rearranged during transfection (*RET*)*,* and *MET*, varies significantly across populations. These variations may affect biomarker expression and interpretation. Nevertheless, despite these limitations, the study provides valuable insights into c-Met protein expression in patients with advanced NSCLC.

In conclusion, data from a cohort of patients with NSCLC treated at City of Hope Medical Center suggest that c-Met protein OE is associated with *MET* mRNA expression and demonstrates limited overlap with other *MET* aberrations. In this real-world cohort, c-Met protein OE was also associated with poorer prognosis. Concordance of c-Met protein OE with *MET* mRNA expression levels was observed. Ongoing research into the molecular characterization of c-Met protein–overexpressing NSCLC and associated biomarkers may pave the way for targeted therapies in patients who lack specific genomic alterations.

## Supplementary Material



## Data sharing statement

AbbVie is committed to responsible data sharing regarding the clinical trials we sponsor. This includes access to anonymized, individual, and trial-level data (analysis data sets), as well as other information (e.g. protocols, clinical study reports, synopses, or statistical analysis plans), as long as the trials are not part of an ongoing or planned regulatory submission.

These clinical trial data can be requested by any qualified researchers who engage in rigorous, independent, scientific research and will be provided following review and approval of a research proposal, Statistical Analysis Plan (SAP), and execution of a Data Use Agreement (DUA). Data requests can be submitted at any time after approval in the US and Europe and after acceptance of this manuscript for publication. The data will be accessible for 12 months, with possible extensions considered. For more information on the process or to submit a request, visit the following link: https://vivli.org/ourmember/abbvie/ then select ‘Home’.
