# Supplementary material for: Prevalence, molecular characterization, and prognosis of c-Met protein overexpression in a real-world cohort of patients with non-squamous non-small cell lung cancer
Source: Acta Oncol. 2025 Nov 18;64:44344. doi: 10.2340/1651-226X.2025.44344 (PMC12640106; doi:10.2340/1651-226X.2025.44344)
Supplement: Supplementary file 1 [file AO-64-44344-s1.pdf]

Supplementary material has been published as submitted. It has not been copyedited, or typeset by Acta Oncologica

Supplemental Material

Supplemental Data

Figure 1. Overlap of *MET* and *TROP2* mRNA levels as measured by RNA-Seq in PD-L1–high, –low, and –negative tumor specimens among patients with NSCLC. *MET* and *TROP2* mRNA levels presented as log2*(expression). PD-L1 high: ≥50% cells expressing PD-L1; PD-L1 low: 1–49% cells expressing PD-L1; PD-L1 negative: ≤1% cells expressing PD-L1. NSCLC, non-small cell lung cancer; PD-L1, programmed-death ligand 1; RNA-Seq, RNA sequencing; RPKM, reads per kilobase per million reads; TROP2, trophoblast cell-surface antigen 2.

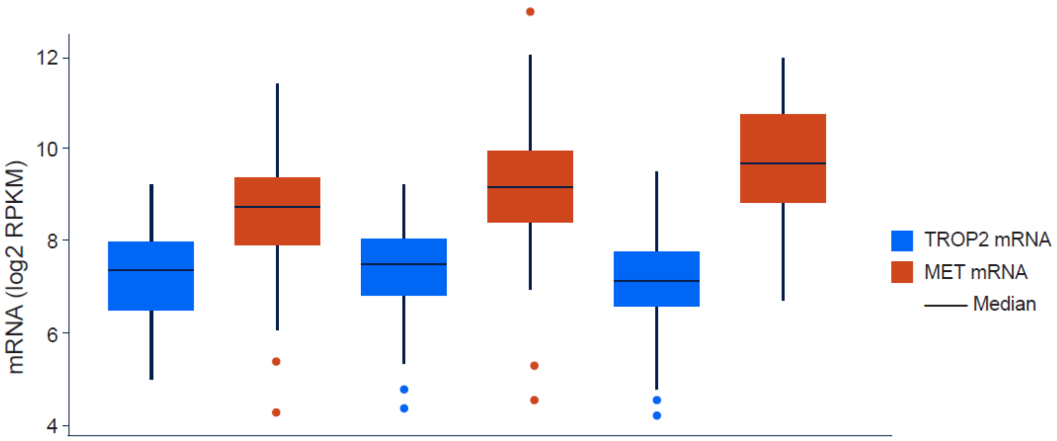

	PD-L1 Negative		PD-L1 Low		PD-L1 High	
	TROP2 mRNA	MET mRNA	TROP2 mRNA	MET mRNA	TROP2 mRNA	MET mRNA
Patient Samples, n	88	88	52	52	23	23
Median	7.4	8.7	7.5	9.2	7.1	9.7
